# Diverse Epigenetic Regulations of Macrophages in Atherosclerosis

**DOI:** 10.3389/fcvm.2022.868788

**Published:** 2022-03-29

**Authors:** Hongmei Yang, Yue Sun, Qingchao Li, Fengyan Jin, Yun Dai

**Affiliations:** ^1^Laboratory of Cancer Precision Medicine, The First Hospital of Jilin University, Changchun, China; ^2^Department of Critical Care Medicine, The First Hospital of Jilin University, Changchun, China; ^3^Department of Hematology, The First Hospital of Jilin University, Changchun, China

**Keywords:** macrophage, polarization, epigenetic, reprogramming, chronic inflammation, atherosclerosis, therapeutic target

## Abstract

Emerging research on epigenetics has resulted in many novel discoveries in atherosclerosis (AS), an inflammaging-associated disease characterized by chronic inflammation primarily driven by macrophages. The bulk of evidence has demonstrated the central role of epigenetic machinery in macrophage polarization to pro- (M1-like) or anti-inflammatory (M2-like) phenotype. An increasing number of epigenetic alterations and their modifiers involved in reprogramming macrophages by regulating DNA methylation or histone modifications (e.g., methylation, acetylation, and recently lactylation) have been identified. They may act to determine or skew the direction of macrophage polarization in AS lesions, thereby representing a promising target. Here we describe the current understanding of the epigenetic machinery involving macrophage polarization, to shed light on chronic inflammation-driving onset and progression of inflammaging-associated diseases, using AS as a prototypic example, and discuss the challenge for developing effective therapies targeting the epigenetic modifiers against these diseases, particularly highlighting a potential strategy based on epigenetically-governed repolarization from M1-like to M2-like phenotype.

## Introduction

Atherosclerosis (AS) is known as one of inflammaging-associated diseases (IAADs) that includes more than 100 different diseases involving almost all human organs (1). AS-related acute cardiovascular or cerebrovascular events (e.g., heart attack and ischemic stroke) currently represent the leading causes of death worldwide (2). The common feature of IAADs is sustained inflammation due to impaired transformation from inflammation into resolution, an event driven primarily by the disproportionate polarization of macrophages (3–5). For example, as the most prevalent type of immune cells in AS lesions, macrophages play an essential role in orchestrating the entire process of AS till plaque rupture (4), thus named AS-associated macrophages (ASAM). High plasticity enables macrophages to polarize toward pro- (M1-like) and anti-inflammatory (M2-like) phenotype in response to different environmental cues (6), which is controlled at the transcriptional level *via* epigenetic modifications (known as marks or codes) of DNA and histones regulated by various epigenetic modifiers (7), including writers that add epigenetic marks onto DNA or histones, erasers that remove epigenetic marks from DNA or histones, and readers that recognize epigenetic marks and facilitate gene transcription. Despite recent discoveries on the essential roles of epigenetics in inflammation and immunity, the functional epigenetic investigation in IAADs is just emerging (8). Here we present the current understanding of the epigenetic mechanisms regulating macrophage polarization and functions, and discuss a novel strategy targeting epigenetic machinery for macrophage repolarization in IAADs such as AS. To avoid the confusion about the terms of various macrophage phenotypes due to the diversity of nomenclature (9), the terms M1 and M2, unless otherwise specified, used in this article only refer to macrophages with pro-inflammatory and anti-inflammatory/pro-resolving properties, respectively.

## Atherosclerosis – A Disease Driven by Non-Resolving Inflammation

A widely accepted concept is that M1-like ASAMs play a pro-AS role by initiating and accelerating inflammation; by contrast, M2-like ASAMs act against AS by stopping inflammation and promoting resolution. Moreover, M1-like ASAMs promote necrosis and thin the protective fibrous cap by secreting proteases (e.g., matrix metallopeptidases such as MMP2 and MMP9), leading to plaque rupture and acute thrombosis. M2-like ASAMs remove apoptotic or dead cells through efferocytosis, produce collagen to thicken the fibrous cap, and secrete growth factors to promote tissue repair, thereby facilitating plaque stabilization or even regression (10). Indeed, M1-like ASAMs are often found in the rupture-prone plaques, while M2-like ASAMs usually appear in more stable plaques and away from the lipid core of the lesions (11). AS patients display a heavily-imbalanced ratio of M1-like to M2-like phenotype, which correlates with disease severity (12). The concept that AS is an inflammatory disease has further been supported by the recent identification of various inflammatory (M1-like) phenotypes of ASAMs at single-cell level in murine and human AS plaques (13–15). However, current anti-AS therapies, including cholesterol-lowering agents (e.g., statins), angiotension-converting enzyme inhibitors, β-blockers, and aspirin, have little direct effect on macrophage polarization because they do not specifically target macrophages.

The principle behind the development of an effective anti-AS therapy is to reduce and stabilize AS lesions to prevent disease progression and fatal complications such as myocardial infarction (MI) and stroke. Over the past decades, tremendous efforts have been made in developing anti-inflammatory therapy to reduce inflammation (16), including blocking inflammatory cell recruitment (e.g., by antagonists of chemokine receptors or adhesion molecules) (17), stabilizing plaques (e.g., by inhibitors of MMPs), and neutralizing pro-inflammatory factors (e.g., by monoclonal antibodies against various cytokines or chemokines) (18). However, almost all of them have failed at preclinical or early clinical phases. Notably, a large randomized trial (named CANTOS) involving more than 10,000 patients with previous MI and high C-reactive protein levels has shown that the IL-1β monoclonal antibody canakinumab could reduce C-reactive protein levels and the incidence rate of recurrent cardiovascular events, without affecting the LDL cholesterol level, but no significant difference in all-cause mortality, probably due to increased risk of fatal infection and sepsis (19). Thus, although CANTOS has, for the first time, approved the “inflammatory hypothesis” of AS in a clinical setting, IL-1β seems not an ideal target (20). Of note, these approaches mainly neutralize various pro-inflammatory factors (e.g., cytokines, chemokines) secreted by inflammatory cells (e.g., macrophages) rather than block their production. Another important lesson learned from the previous failures is that an effective anti-inflammatory approach may rely on both inflammation inhibition and resolution promotion, which require not only the termination of inflammatory cell recruitment and activation (reducing M1-like macrophages) but also the removal of apoptotic or dead cells *via* efferocytosis (increasing M2-like macrophages) (21).

Plaque regression is clinically desirable, which may be achieved by redirecting ASAM polarization from M1-like to M2-like phenotype (10, 22). For example, increasing HDL levels by targeting miR-33 (an intronic microRNA located within the *SREBF2* gene that encodes sterol regulatory element binding transcription factor 2) leads to the regression of established plaques in *Ldlr*−/− mice (23). Silencing of *IRF5* (encoding interferon regulatory factor 5) reprograms ASAMs from M1-like to M2-like phenotype and improves post-MI healing by augmenting resolution (24). Whereas the deficiency of efferocytosis due to high levels of the “don’t eat me” signal CD47 is observed in advanced plaques, CD47-blocking antibodies significantly prevent disease progression by restoring the phagocytotic capability of M2-like ASAMs (25). Taken together, these findings strongly argue that the therapeutic strategy promoting phenotypic transition from M1-like to M2-like phenotype may effectively attenuate and even block non-resolving inflammation, delaying or halting AS progression or leading to plaque regression.

## Macrophage Repolarization – A Potential Approach for Reprogramming Macrophages From Pro- to Anti-Inflammatory Phenotypes

Traditionally, macrophages can polarize toward classically activated M1 [e.g., M(IFNγ), M(LPS), and M(LPS + IFNγ)] and alternatively activated M2 [e.g., M(IL-4)] phenotype upon different stimuli (9, 26). While recent application of single-cell RNA sequencing (scRNAseq) has revealed high heterogeneity of ASAMs in AS plaques (13–15), the theme of macrophage polarization seems not to have been changed. However, the classical model for macrophage polarization may be challenged by our genome-wide survey based on a published public available database (27). By comparing the gene expression profiling (GEP) between resting (M0) and M1 [M(LPS + IFNγ)] or M2 [M(IL-4)] macrophages, we have noted some interesting phenomena in gene expression reprogramming during macrophage polarization, including a) that a majority of changes in differentially expressed genes (DEGs, including up- and down-regulated ones) occur during M1 [M(LPS + IFNγ)] polarization (versus M0, particularly involving pro- and anti-inflammatory genes as well as the genes involved in inflammatory pathways), while most of these changes (>80%) are, however, reversed in M2 or M(IL-4) [versus M1 or M(LPS + IFNγ)]; in contrast, much less DEGs (only 16 genes) was observed during M2 or M(IL-4) polarization (versus M0); b) that the DEGs in M1 [M(LPS + IFNγ)] polarization but reversed in M2 [M(IL-4)] are functionally enriched for several key pathways (e.g., proteasome, NF-κB, JAK/STAT, and apoptosis for the up-regulated DEGs; lysosome, oxidative phosphorylation, and PPAR for the down-regulated DEGs); and c) interestingly, these pathways appear to be shared by differentiation of monocytes into macrophages.

Accordingly, we hypothesize an alternative model for macrophage polarization, in which in addition to the classical models (i.e., polarization of M0 to either M1 or M2 upon different stimuli), M1 may be skewed directly to M2 simply by turning off the M1 program, a process termed repolarization, similar to inter-phenotypic transition (or trans-differentiation) in the epigenetic landscape originally described by Waddington and later updated by many others (28). According to this model, non-resolving inflammation in AS lesions might be caused by deficient repolarization of ASAMs (4). Therefore, targeting the machinery that governs the repolarization from M1-like to M2-like phenotype provides a rationale for developing an effective “double-hit” anti-inflammatory therapy, which simultaneously inhibits inflammation (reducing M1-like phenotype) and promotes resolution (increasing M2-like phenotype). In this context, the mechanism driving macrophage repolarization may be associated with histone lactylation, a novel form of epigenetic modification, which occurs in the late stage of M1 [M(LPS + IFNγ)] and is related to M2 gene expression (29). Interestingly, histone lactylation in macrophages is induced primarily by lactate, an “end-product” of glycolysis, which accumulates during M1 polarization due to a metabolic paradigm shift from oxidative phosphorylation to glycolysis (30).

## Epigenetic Modifying Enzymes – An Expanding Superfamily Governing Hierarchical Reprogramming of Macrophages

Epigenetics is defined as the coding of gene expression in a highly tissue/cell- and context-specific manner *via* modifications of DNA and histones without altering the DNA sequence itself (8). The transition of cell phenotypes and maintenance of cell identity are controlled by the epigenetic machinery through reprogramming gene expression at the transcriptional level (31). To date, epigenetics has been studied in the context of chromatin modifications in either DNA or histones, and recently in higher-order chromatin structures involving large epigenomic domains named lamina-associated domains (LADs) and large, organized chromatin lysine modifications (LOCKs) (32). Epigenetic modifications, often called codes or marks, include DNA methylation of the nucleotide cytosine (e.g., 5mC and 5hmC, 5fC, 5caC, 3mC, and 6mA) at CpG sites and histone post-translational modifications [PTMs e.g., methylation, acetylation, phosphorylation, ubiquitylation, sumoylation, butyrylation, formylation, propionylation, citrullination, crotonylation, proline isomerization, ADP ribosylation, succinylation, 2-hydroxy isobutylylation, and more recently lactylation (29)] mostly at lysine residues (33). Other epigenetic mechanisms include various RNA modifications (e.g., m6A, m5C, m1A, 2’-O-Me, and Ψ) and non-coding RNAs (e.g., lncRNA, microRNA, and circRNA). DNA methylation is regulated by DNA methyltransferases (e.g., DNMT1, DNMT2, DNMT3A, and DNMT3B) and ten-eleven translocation methylcytosine dioxygenases (e.g., TET1, TET2, and TET3). The most common histone PTMs are lysine methylation and acetylation, which are reciprocally regulated by two classes of histone-modifying enzymes (i.e., “writer” and “eraser”). The writer that adds epigenetic codes to specific residues of histones includes lysine methyltransferase (KMT) and histone acetyltransferase (HAT). The eraser that removes these codes includes lysine demethylase (KDM) and histone deacetylase (HDAC). Histone PTMs result in a “loose” (open) or “tight” (closed) chromatin configuration, called chromatin remodeling, which control the accessibility of transcriptional factors to the promoter or enhancer regions of target genes on DNA, thereby triggering or silencing their expression.

Another category of epigenetic molecules called “reader” that recognizes epigenetic codes and recruit transcription-regulatory factors to target genes includes two families i.e., bromodomain and extraterminal protein (BET) and malignant brain tumor domain protein (MBT) (31). BETs read histone acetylation codes through their distinct bromodomain (BRD) and then recruit positive transcription elongation factor b (P-TEFb, a complex of CDK9 and cyclin T), which in turn phosphorylates the C-terminal domain (CTD) of RNA polymerase II to trigger transcription initiation and elongation (34). MBTs (e.g., MBT, chromodomain, tudor domain) recognize histone methylation codes, but their role in macrophages remains unknown. Taking the advantage of recent advances in the development of specific BRD inhibitors in cancer treatment, these agents may also emerge as a potential therapy for IAADs such as AS.

An additional category of epigenetic molecules called chromatin remodeler (or nucleosome remodeling factor, NURF) includes at least four subfamilies: switch/sucrose non-fermenting (SWI/SNF), imitation switch (ISWI), inositol requiring 80-like (INO80-like) and chromodomain helicase DNA binding (CHD) (35). The remodelers are recruited to their target regions by transcription factors (TFs) or non-coding RNAs and forms the ATP-dependent chromatin remodeling complexes, which facilitate transcription precisely and accurately in time and space, *via* multiple mechanisms including nucleosome-positioning or nucleosome sliding, creation of a remodeled state for DNA to be more accessible with histones still bound, altering histone–DNA interactions, disassembly of nucleosomes, exchange of histones with variants of different properties, and regulation of higher-order chromatin structures. Although the role of remodelers in macrophages remains unknown, the characterization of their structures may pave an entirely new avenue for drug development to treat various diseases (36, 37).

With further understanding their functions, all epigenetic-regulatory proteins have been re-categorized into three classes, including epigenetic modifiers that directly control DNA methylation (e.g., DNMTs and TETs), histone PTMs (e.g., KMTs and KDMs for methylation and HATs and HDACs for acetylation), or higher-order chromatin structures; epigenetic mediators that serve as the downstream targets of epigenetic modifiers and in turn govern cell plasticity and phenotypes *via* reprogramming; and epigenetic modulators that regulate the activity or subcellular localization of epigenetic modifiers and bridge (micro)environment with epigenomics (25).

## Epigenetic Alterations – Emerging Evidence Supporting Atherosclerosis as an “Epigenetic” Disease

Fast-accumulating evidence indicates the correlation between epigenetic alterations and the risk of AS, including DNA methylation and its regulatory enzymes (e.g., DNMTs and TETs), as well as histone PTMs and their writers and erasers. To date, at least 15 types of histone PTMs and more than 130 sites have been identified, which regulate gene expression *via* chromatin remodeling (33). Functionally, histone PTMs (particularly methylation and acetylation, mostly involving histones H3 and H4) can be divided into activating and inhibitory types, which either allow or prevent the transcription of target genes during cell phenotype transition like macrophage polarization. In general, histone lysine (K) acetylation often activates gene expression *via* increasing the accessibility of TFs, which is reciprocally regulated by specific HATs and HDACs. However, histone lysine methylation can be either activating or inhibitory in regulating the transcription of target genes, dependently on which lysine residue(s) is methylated with how many methyl groups, which is reciprocally regulated by KMTs and KDMs. Among numerous epigenetic modifiers identified to regulate histone PTMs, only a small number of them have been identified in the regulation of macrophage polarization and functions thus far.

### DNA Methylation

#### Alterations of DNA Methylation in AS-Associated Macrophages

DNA methylation involves the transfer of a methyl group to the C5 position of the cytosine to form 5mC, an event mediated by DNMTs, which primarily silence the expression of target genes by preventing the binding of TFs to DNA. DNA demethylation is mediated by TETs, which catalyzes oxidation of 5mC to 5hmC, 5fC, and then 5caC, thereby removing this inhibitory epigenetic mark to allow gene transcription. In AS, DNA methylation is specifically associated with disease type and progression, as well as disease onset, vascular events, and plaque stabilization. For example, there is a unique whole-genome landscape of DNA methylation in AS lesions, compared to surrounding normal vessel tissue (38); a disease- or location-specific methylation pattern is associated with MI, rather than ischemic stroke (39); disease progression-specific CpG methylation profiles correlate with the grade of lesions (40); after cerebrovascular events, global demethylation is associated with up-regulation of anti-inflammatory genes and likely contributes to plaque stabilization (41). Moreover, DNA methylation of specific target genes (e.g., *TRAF3* that encodes TNF receptor-associated factor 3, *PPM1A* that encodes protein phosphatase 1A, among many others) is also closely associated with AS or its treatment (42). With rapid advances in the technology of single-cell analysis such as scRNAseq and mass cytometry by the time-of-flight (CyToF) (43, 44), cell type-specific profiles of either global or gene-specific DNA methylomes (e.g., macrophage, endothelial cell/EC, smooth muscle cells/SMC, lymphocyte, etc.) may soon be available for more precisely monitoring the dynamic changes of DNA methylation as well as understanding their clinical significance in AS.

#### The Roles of DNA Methylation Modifiers in AS-Associated Macrophages

To date, understanding of the functional roles of the epigenetic modifiers involving DNA methylation is relatively limited in AS. Functionally, DNMT3A and DNMT3B catalyze *de novo* DNA methylation. DNMT3A and TET2 represent the most commonly mutated genes in patients with coronary heart disease carrying clonal hematopoiesis of indeterminate potential (CHIP) (45). Transplantation with bone marrow of *Dnmt3a*−/− mice significantly increases plaque size of *Ldlr*−/− mice (46), suggesting the anti-AS role of DNMT3A. However, *Dnmt3a* expression is suppressed in M2 [M(IL-4)] macrophages, probably *via* an lncRNA called DNMT3aos located on the antisense strand of *Dnmt3a* (47). Interestingly, the effect of TET2 resembles that for DNMT3A, although these two epigenetic enzymes have opposite functions in regulating DNA methylation. Partial reconstitution of bone marrow clonal hematopoiesis by transplanting *Tet2*-mutant cells increases plaque size in *Ldlr*−/− mice, in association with increased IL-1β production by *Tet2*-mutant ASAMs *via* NLRP3 inflammasome (48). TET2 specifically represses IL-6 expression in macrophages, an event for inflammation resolution (48, 49). DNMT1 is a DNA methyltransferase maintaining DNA methylation. Macrophage-specific expression of *Dnmt1* promotes AS in *Apoe*−/− mice, *via* increasing M1 cytokine production but suppressing the M2 gene expression, in association with promoter methylation and thus down-regulation of PPAR-γ or KLF4 (Kruppel-like factor 4) (50, 51). Following the paradigm shift from profiling epigenomics to functional epigenetics in the epigenetics field (52), the functions of these or other epigenetic enzymes involving DNA methylation will be defined more precisely and thus become a potential targets for anti-inflammatory therapy.

### Histone Methylation

#### The Roles of Lysine Methyltransferases in AS-Associated Macrophages

**FIGURE 1:**
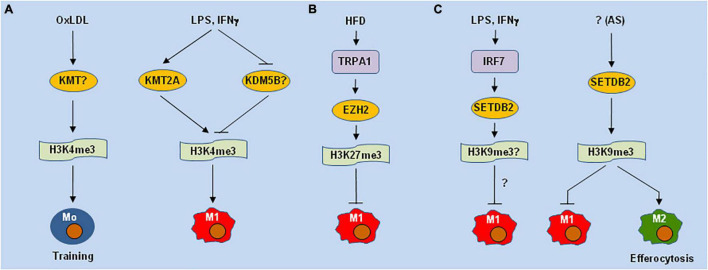
Different lysine methyltransferases may play opposite roles of in macrophage polarization and functions. **(A)** H3K4me3 mediated by lysine methyltransferases (KMTs e.g., KMT2A/MLL) is involved in oxLDL-induced training immunity of monocytes (Mo) in atherosclerosis (AS) and LPS-induced M1 polarization. **(B)** High-fat diet (HFD) induces the expression of TRPA1 (a calcium permeable non-selective cation channel), which stabilizes EZH2 (PRC2 or KMT6) to inhibit M1 polarization *via* H3K27me3. **(C)** LPS and IFNs up-regulates SETDB2 *via* IRF7, while deletion of SETDB2 in hematopoietic cells promotes M1-like polarization and impairs efferocytosis, a function of M2 macrophages, *via* H3K9me3 in AS lesions. oxLDL, oxidized low-density lipoprotein; LPS, lipopolysaccharide; TRPA1, transient receptor potential ankyrin 1; EZH2, enhancer of zeste homolog 2; IFN, interferon; IRF7, interferon regulatory factor 7; SETDB2, SET domain bifurcated histone lysine methyltransferase 2.

H3K4me3 is one of activating histone PTMs, which is reciprocally regulated by MLL (KMT2A) and KDM5B, respectively. Emerging evidence supports the involvement of H3K4me3 in the regulation of macrophages involving AS. H3K4 methylation stepwisely increases in macrophages during AS progression, in association with disease severity (53). Training monocytes by oxLDL, rather than native LDL, results in up-regulation of multiple pro-inflammatory and pro-AS genes, in association with H3K4me3 on their promoters (Figure 1A), while this event can be abolished *via* H3K4me3 inhibition by pan-KMT inhibitors (54). MLL is up-regulated in M1 [e.g., M(IFNγ)] macrophages, in association with increased H3K4me3 (Figure 1A); in contrast, its enzyme activity is reduced in M2 [M(IL-4 + IL13)] macrophages (55). Increased H3K4me3 up-regulates the pro-inflammatory genes, which can be prevented by an inhibitor of the MLL-Menin interaction (MI2-2). Consistently, pan-KMT inhibition (e.g., by 5’-methylthioadenosine/MTA) also inhibits LPS-induced expression of M1 genes and LPS + IFNγ-triggered secretions of pro-inflammatory cytokines (56).

H3K9me3 and H3K27me3 represent two inhibitory PTMs. Both of these PTMs are markedly increased in ASAMs and lymphocytes in AS plaques, but they are undetectable in healthy vessel tissues (57). However, these PTMs markedly decrease during disease progression (53), while they do not correlate with the expression of their KMTs, including PRC2 (EZH2 or KMT6, which inhibits M1-like polarization in AS lesions (58)) (Figure 1B), the H3K27me3 methyltransferase G9a (KMT1C), and the H3K9me3 methyltransferases SETDB1 (KMT1E) or SUV39H1/2 (KMT1A/B). SETDB2, a member of the KMT1 family that methylates H3K9, is up-regulated in M1 [M(LPS)] but not M2 [M(IL-4)] macrophages (59). While SETDB2 is highly expressed in murine AS lesions, its genetic deletion in hematopoietic cells promotes inflammation and accelerates AS in *Ldlr*−/− mice, in association with enhanced expression of pro-inflammatory genes but attenuated efferocytosis in CD45^+^ ASAMs (Figure 1C).

#### The Roles of Lysine Demethylases in Macrophage Polarization and AS-Associated Macrophages

**FIGURE 2:**
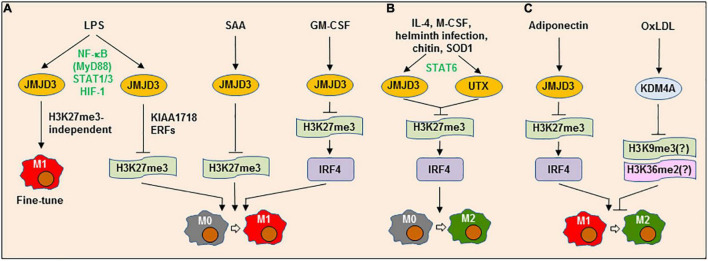
Lysine demethylases can play dual roles in both M1 and M2 polarization or M1→M2 repolarization. **(A)** LPS up-regulates the lysine demethylase (KDM) jumonji domain-containing (JMJD3) (KDM6B) *via* a process involving multiple transcription factors (e.g., NF-κB/MyD88, STAT1/3, and HIF-1), which in turn acts to either fine-tune M1 phenotype *via* an H3K27me3-independent process or promote M1 polarization, which requires another KDM KIAA1718 and elongation-regulatory factors (ERFs). Similarly, JMJD3 is involved in M1-like polarization induced by serum amyloid A (SAA) or granulocyte-macrophage colony-stimulating factor (GM-CSF) by up-regulating IRF4 *via* H3K27me3. **(B)** Alternatively, multiple factors (e.g., IL-4, M-CSF, helminth infection, chitin, and SOD1) can induce JMJD3 and UTX (KDM6A) expression *via* STAT6, which in turn mediate M2 polarization by up-regulating IRF4 *via* H3K27me3. **(C)** JMJD3 may promote adiponectin-triggered repolarization from M1-like to M2-like phenotype, likely *via* the similar mechanism involving H3K27me3-depdendent IRF4 expression. On the contrary, oxLDL induces the expression of KDM4A (JMJD2A), another member of the JMJD demethylase family, which blocks the repolarization from M1-like to M2-like phenotype, an event that can be restored by KDM4A deficiency or inhibition. LPS, lipopolysaccharide; JMJD3, Jumonji domain-containing protein 3; NF-κB, nuclear factor kappa-light-chain-enhancer of activated B cells; STAT, signal transducer and activator of transcription; HIF-1, hypoxia-inducible factor 1; IRF4, interferon regulatory factor 4; IL-4, interleukin-4; M-CSF, macrophage colony-stimulating factor; SOD1, superoxide dismutase 1; oxLDL, oxidized low-density lipoprotein; KDM4A, lysine demethylase 4A.

Jumonji domain-containing 3 (JMJD3) (KDM6B), which specifically demethylates H3K27me3, is involved in the reprogramming of M1 [M(LPS)] polarization (60) (Figure 2A). More than 70% of LPS-inducible genes are JMJD3 targets in macrophages. However, although *Jmjd3* deletion increases H3K27me3, but does not markedly affect most of those LPS-induced genes, suggesting that JMJD3 only acts to fine-tune the transcriptional program for inflammatory gene expression in LPS-activated macrophages and this action is independent of its H3K27me3 demethylase function. Although the enzymatic activity of JMJD3 seems unable to determine the direction of macrophage polarization by itself, it works together with the H3K9me2/H3K27me2/H4K20me1-specific demethylase KIAA1718 and certain transcription elongation-regulated proteins to demethylate H3K27me3 of pro-inflammatory genes. In contrast, down-regulation of either JMJD3 or KIAA1718 attenuates mRNA elongation of these genes (61). In this case, JMJD3 may require collaboration with an additional epigenetic modifier (e.g., KIAA1718) to act as an H3K27me3 demethylase in M1 reprogramming, at least in response to LPS. Serum amyloid A (SAA), another pro-inflammatory factor involved in AS, induces JMJD3 expression, in association with reduced H3K27me3 in macrophages (62). Unlike LPS-induced genes, SAA-induced expression of pro-inflammatory genes can, however, be blocked by *Jmjd3* knockdown or inactivation, *via* restoration of H3K27me3, suggesting that the JMJD3 functions may vary in a stimulus-specific manner. Considering JMJD3 and H3K27me3 as a potential target in inflammation (63), the selective inhibitors of H3K27-specific demethylases have been developed as a novel anti-inflammatory therapy. For example, GSK-J1 (an inhibitor of JMJD3 and UTX, both belonging to the KDM6 subfamily), which specifically binds to the catalytic pocket of JMJD3 to inhibit its demethylase activity, sharply inhibits pro-inflammatory gene expression in macrophages, *via* increasing H3K27me3 (64).

On the other hand, IL-4 also induces JMJD3 expression and thus activates M2 genes by reducing H3K27me2/3 at their promoters (65), suggesting a role of JMJD3 in M2 [e.g., M(IL-4)] polarization (Figure 2B). JMJD3 also mediates M2-like polarization in response to M-CSF, but is not involved in M1-like polarization induced by GM-CSF. In this context, helminth infection or chitin fails to induce M2-like polarization in *Jmjd3*−/− mice (66). Although *Jmjd3* deficiency results in a global increase in H3K27me3 at the promoters of numerous genes, only a small number of them are specifically affected by *Jmjd3* deletion. In the latter, IRF4 (interferon regulatory factor 4) represents one downstream target responsible for M2-like polarization, expression of which is associated with the demethylase activity of JMJD3. While *Irf4* knockout copies the phenotype of *Jmjd3* deletion, restoration of IRF4 expression can rescue the M2-like polarization that is impaired in *Jmjd3*−/− macrophages. However, GM-CSF also induces IRF4 expression *via* enhancing JMJD3 demethylase activity, leading to the production of pro-inflammatory CCL17 in a murine model of arthritis, an event blocked by GSK-J1 (67). In addition, the JMJD3-IRF4 axis may also contribute to repolarization of M1-like to M2-like phenotype mediated by adiponectin (68) (Figure 2C). Therefore, JMJD3 and its downstream targets such as IRF4 play dual functions context-specifically in both M1 and M2 polarization.

JMJD3 expression is regulated at transcriptional level by a number of TFs. LPS induces JMJD3 expression *via* a MyD88-dependent activation of the NF-κB pathway in ECs (69) or the activation of STAT1 and STAT3. For the latter, knockdown of both *Stat1* and *Stat3*, rather than either of them, inhibits the expression of JMJD3 and pro-inflammatory genes in microglia (70). As one of the HIF-1-dependent genes (71), JMJD3 expression also involves the HIF pathway in response to LPS (72). Whereas IL-4 induces JMJD3 expression *via* STAT6 activation in macrophages, *Stat6* knockout only prevents JMJD3 expression induced by IL-4, but not LPS (65). Moreover, *Stat6* deficiency prevents H3K27me3 demethylation and M2 gene expression in response to IL-4, while has no effect on the pro-inflammatory effect of LPS. Similar phenomenon has also been found in SOD1-induced M2-like polarization (73). Thus, although the roles of JMJD3 and its downstream targets (e.g., IRF4) may not be specific to M1 or M2 polarization, the upstream signals (e.g., diverse TFs) of JMJD3 seem to determine the direction of JMJD3-mediated macrophage polarization (74). Of note, myeloid *Jmjd3* deficiency leads to progression of AS lesions in *Ldlr*−/− mice, suggesting an anti-AS role of JMJD3 in ASAMs (75). However, it remains unclear whether JMJD3 acts to promote the polarization of ASAMs toward an anti-inflammatory (M2-like) phenotype in this setting.

KDM4A [JMJD2A or jumonji C-domain-containing histone demethylase 3A (JHDM3A)] demethylates H3K9me3, H3K36me2, and H1.4K26me3. We have identified KDM4A as another member of the JMJD demethylase family that regulates M1-like polarization, at least in response to oxidized low-density lipoprotein (oxLDL) (76) (Figure 2C). Exposure to oxLDL results in KDM4A up-regulation, accompanied by the expression of multiple M1 genes. While *KDM4A* knockdown prevents oxLDL-induced M1-like polarization, it instead promotes the expression of M2 genes. As an oxygen sensor, hypoxia increases the protein level of KDM4A, but not its mRNA level (77), by preventing its degradation *via* the SCF-containing ubiquitin ligase complex (78). In contrast, other JMJD family members (e.g., JMJD3) are primarily regulated at the transcriptional level *via* HIF-1α activation (79). However, although oxLDL also activates the NF-κB and HIF pathways (80), KDM4A expression seems to be independent of the NF-κB and HIF-1 pathways in macrophages exposed to oxLDL (76). Therefore, KDM4A inhibition may skew ASAM polarization straight from M1-like to M2-like phenotype (i.e., repolarization), thus serving as a potential target for the development of anti-inflammatory therapy against AS.

#### The Role of Histone Methylation in Trained Immunity Involving Atherosclerosis

Traditionally, the epigenetic modifications were considered to act only temporarily due to their reversible features and thus not to contribute to immune memory. However, this concept has been challenged by recent discoveries that immune cells (e.g., macrophages) can memorize the cellular states and perturbations (e.g., environmental stimuli) without changing the DNA sequence. Such an epigenetic memory is able to burst a rapid and robust inflammatory response once cells encounter the same or even different stimuli (8). For example, Western diets can cause NLRP3-dependent immune memory in monocytes/macrophages, which contributes to chronic and stepwisely worsening inflammation in AS plaques (81). Later, such phenomenon has been attributed to trained immunity (e.g., by oxLDL) *via* histone epigenetic modifications (e.g., H3K4me1 and H3K4me3), which could be prevented by the inhibitors of HMTs (54, 82, 83). Of note, trained immunity driven by epigenetic reprogramming is closely associated with metabolic rewiring from oxidative phosphorylation to glycolysis (84–86), a well-documented hallmark of M1 macrophages. However, which HMT(s) or KDM(s) are responsible for trained immunity remains to be defined.

### Histone Acetylation

#### The Roles of Histone Acetyltransferases in Macrophage Polarization

Histone acetylations are generally activating PTMs that promote the expression of target genes. To date, only a few HATs have been identified to be involved in macrophage polarization. EP300 (KAT3B) binds to c-Myc *via* protein arginine methyltransferase 1 (PRMT1) and is recruited to the promoters of target genes, resulting in M2 gene expression (87). Another HAT CBP (KAT3A) mediates transcriptional activation of IFNβ *via* increasing H3K56-Ac (88). However, a novel HAT named MOF (KAT8) promotes TNF-α/NF-κB-mediated expression of pro-inflammatory genes *via* increasing H4K16-Ac (89).

#### The Roles of Histone Deacetylases in Macrophage Polarization and AS-Associated Macrophages

**FIGURE 3:**
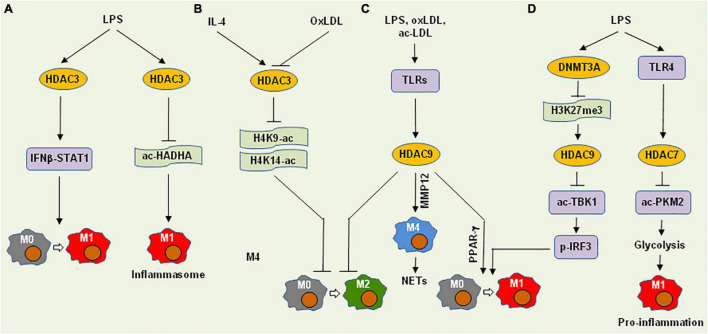
Histone deacetylases promote M1 polarization and pro-inflammatory functions of macrophages *via* deacetylation of histones or non-histone proteins. **(A)** HDAC3 mediates LPS-induced M1 polarization and inflammasome activation *via* the IFNβ-STAT1 signaling cascade and HADHA deacetylation that restricts fatty acid oxidation, respectively. **(B)** On the other hand, HDAC3 can block M2 polarization involving either IL-4 or oxLDL *via* deacetylation of H4K9-ac and H4K14-ac. **(C)** Multiple pro-inflammatory factors (e.g., LPS, oxLDL, and ac-LDL) induce the expression of HDAC9 *via* toll-like receptors (TLR), which in turn inhibits M2 polarization, promotes M1 polarization *via* PPARγ, or induces M4 macrophages (in conjunction with MMP12) that recruit neutrophils to form neutrophil extracellular traps (NETs). **(D)** LPS induces HDAC9 expression *via* DNMT3A-associated repression of H3K27me3, thus triggering M1 polarization *via* deacetylation of TBK1 that phosphorylates IRF3. Alternatively, LPS up-regulates HDAC7 *via* TLR4, which deacetylates PKM2 to promote glycolysis, thereby promoting pro-inflammatory function of M1 macrophages. HDAC, histone deacetylase; IFNβ, interferon β; STAT1, signal transducer and activator of transcription 1; HADHA, hydroxyacyl-CoA dehydrogenase trifunctional multienzyme complex subunit alpha; oxLDL, oxidized low-density lipoprotein; ac-LDL, acetylated low-density lipoprotein; MMP12, matrix metallopeptidase 12; PPARγ, peroxisome proliferator-activated receptor gamma; DNMT3A, DNA methyltransferase 3A; TBK1, TANK-binding kinase 1; PKM2, pyruvate kinase muscle isozyme 2; ac-, acetylation; p-, phosphorylation.

HDAC3, a class I HDAC, deacetylates H4K9-Ac and H4K14-Ac, which is involved in the regulation of both M1 and M2 polarization. In HDAC3-deficient macrophages, LPS fails to activate nearly half of the inflammatory genes, suggesting the role of HDAC3 in M1 polarization (90) (Figure 3A). While there are near 700 genomic regions are hyperacetylated at histone H4 in *Hdac3−*/− macrophages, the number of hyperacetylated regions are tripled after LPS stimulation. In contrast, a large number of regions display H4 hypoacetylated in both untreated or LPS-treated *Hdac3−*/− macrophages, in which the recognition motifs for the IRF family and STAT1 are mostly enriched. Moreover, while IRF3 (interferon regulatory factor 3) directly controls *Ifnb1* transcription, the pro-inflammatory IFNβ-STAT1 axis is, however, impaired in *Hdac3−*/− macrophages exposed to LPS, in association with up-regulation of *PTGS1* (encodes COX-1). During NLRP3 inflammasome activation, HDAC3 translocates to mitochondria and thus restricts fatty acid oxidation (FAO) by deacetylating the non-histone protein HADHA (mitochondrial trifunctional protein subunit α) at K303, which reduces its FAO enzyme activity and promotes IL-1β production by shaping mitochondrial adaptation (91). Thus, HDAC3 seems to trigger M1 gene expression indirectly *via* diverse mechanisms.

HDAC3 is also involved in the regulation of M2 polarization (Figure 3B). In this case, *Hdac3−*/− macrophages display an M2-like phenotype analogous to IL-4-induced alternative activation, in association with increased H3K9-Ac and H3K14-Ac (92). Interestingly, although most up-regulated genes in *Hdac3−*/− macrophages can be induced by both IL-4 and LPS in wild-type macrophages, a large number of the down-regulated genes in *Hdac3−*/− macrophages are up-regulated only by LPS in wild-type macrophages. Thus, HDAC3 acts as a suppressor of M2 polarization *via* its deacetylase activity even in the absence of pro-inflammatory stimuli. After exposure to oxLDL, *Hdac3−*/− macrophages secrete more TGF-β, an M2 cytokine, than wild-type counterparts, in association with increased H3K9/14-Ac at the *Tgfb* locus. This suggests that HDAC3 directly binds to the regions near the *Tgfb* promoter and inhibit its expression. Taken together, HDAC3 activation may skew the direction of macrophage polarization from M1-like to M2-like phenotype.

HDAC3 expression is associated with plaque vulnerability in human AS (93), suggesting its pro-AS role. Moreover, conditional knockout of *Hdac3* in macrophages transforms their phenotype to an anti-AS fibrotic phenotype, leading to increased collagen deposition and thus more stable plaques in *Ldlr−*/− mice. Interestingly, while HDAC3 overexpression in ECs promotes endothelial-to-mesenchymal transition (EndMT), HDAC3 inhibition reduces AS lesions in *Ldlr−*/− mice, further supporting the pro-AS role of HDAC3 (94). On the contrary, up-regulation of HDAC3 in ECs may also inhibit inflammation and AS (95). Nevertheless, whether the functions of HDAC3 described above in the regulation of macrophage polarization would be applied in AS remains uncertain.

During the differentiation of monocytes to macrophages, HDAC9 (a class IIa HDAC) are up-regulated (96), which consists of two isoforms with or without HDAC domain, respectively. The latter, named HDAC-related protein (HDRP) or MEF2-interacting transcription repressor protein (MITR), is a truncated form that lacks deacetylase activity, which functions to recruit other HDACs (e.g., HDAC1 or HDAC3). Although HDAC9 is highly expressed in macrophages, it can be further induced by LPS (*via* toll-like receptor, TLR), oxLDL, and acetylated LDL (Figure 3C). High levels of HDAC9 in macrophages is maintained by DNMT3A *via* H3K27me3 repression at it distal promoter region, while HDAC9 in turn binds to TBK1 to enhance its K241 deacetylation and kinase activity (97) (Figure 3D). In this context, *Dnmt3a* deficiency selectively impairs the expression of type I interferons (e.g., IFN-α and IFN-β) induced by LPS, due to inhibition of TBK1-mediated IRF3 phosphorylation. Therefore, HDAC9 could execute its pro-inflammatory functions *via* diverse mechanisms involving multiple upstream and downstream pathways. As relatively high basal levels of HDAC9 seems to be required for macrophages, it would be quite challenging to target only the inducible part of HDAC9 to inhibit inflammation triggered by (micro) environmental stimuli.

A genome-wide association study (GWAS) has unveiled a SNP within the *HDAC9* gene, which is significantly associated with the risk of large vessel stroke (98). Another genome-wide association meta-analysis has identified two genetic loci (*HDAC9* and *RAP1GAP*) associated with aortic calcification, an independent predictor for the risk of cardiovascular events (99). These observations in the large population-based cohorts provide a strong link between HDAC9 and AS. In this context, *Hdac9* deficiency inhibits M1 gene expression *via* up-regulating PPAR-γ, while promotes M2-like polarization of macrophages and expression of the ATP-binding cassette transporter ABCA1 and ABCG1 *via* increasing H3K9-ac at their promoters in *Ldlr−*/− mice (96). In AS plaques, HDAC9 is associated with MMP12 expression in the regions clustered with inflammatory genes in ASAMs. The expression of both *Mmp12* and *Hdac9* is associated with a unique subtype of macrophages named M4 (100), an inflammatory phenotype that recruits neutrophils to form neutrophil extracellular traps (NETs) in response to oxLDL (101). In *Apoe−*/− mice, *Hdac9* deficiency confers plaque stability *via* an alternative mechanism involving deacetylation of the non-histone protein IKKα and IKKβ, which leads to the activation of the NF-κB pathway in both macrophages and ECs to drive inflammatory response (102). Of note, a specific inhibitor of the class IIa HDACs (including HDAC9) limits this pro-inflammatory response and attenuates lesion formation. Thus, due to its dual roles in pro-inflammatory response and plaque vulnerability, HDAC9 is considered as a very promising target for the treatment of AS-related diseases.

HDAC7, another class IIa HDAC, is structurally similar to HDAC9. Unlike the latter, HDAC7, however, has minimal deacetylase activity, while often binds to HDAC3 to suppress gene expression. In pre-B cells, HDAC7 inhibits pro-inflammatory genes essential for macrophage functions, while HDAC7 is specifically down-regulated to release this brake during transdifferentiation of pre-B cells into macrophages (103). In differentiated macrophages, HDAC7 promotes the TLR4-induced expression of a subset of pro-inflammatory genes (Figure 3D), an event prevented by a selective inhibitor of the class IIa HDACs (104). In macrophages, HDAC7 binds to and deacetylates PKM2 (pyruvate kinase muscle isozyme 2) at K433, therefore increasing LPS-induced inflammatory responses *via* promoting glycolysis (105), suggesting a role of HDAC7 in immunometabolism (106). In this case, while the role of HDAC7 in M1 polarization remains uncertain, it appears to be required for maintaining the pro-inflammatory property of M1-like macrophages. Thus, targeting HDAC7 could suppress the pro-inflammatory functions of macrophages, but not reprogramming for M1 polarization. While the role of HDAC7 remains uncertain in AS, it has been demonstrated that HDAC7 plays an important role in the maintenance of vascular integrity by repressing MMP10 expression in ECs (107). The latter would be a major concern for targeting HDAC7 in AS.

The findings involving the effects of HDAC inhibition on inflammation remains controversial thus far. The pan-HDAC inhibitor SAHA (vorinostat) reduces immune cell infiltration and inflammation in AS plaques of hypercholesterolemic *Apoe−*/− mice (108), while another pan-HDAC inhibitor TSA increased macrophage infiltration in AS lesions, in association with increased histone H4 acetylation (109). In macrophages, TSA impairs the expression of most M1 markers and cytokines induced by LPS or IFNγ + LPS, but also inhibits the expression of M2 markers induced by IL-4 (56). In the latter case, TSA down-regulates both basal and IL-4-induced expression of Arg1 *via* an HDAC3-independent process (92). MS-275 (entinostat), a selective inhibitor of class I HDACs (e.g., HDAC1 and HDAC3), increases the basal level of the M2 marker Arg1, similar to the phenotype of *Hdac3* deletion, while does not affect IL-4-induced Arg1 expression.

#### The Roles of Non-histone Acetylation in Macrophages

Numerous non-histone proteins also serve as substrates of deacetylation by HDACs (110), including various TFs (e.g., RelA/p65, p53, STAT1). For example, the activation of the NF-κB pathway represents a major signal required by M1 [M(IFNγ)] polarization and functions, while NF-κB inhibition can promote M2 [M(IL-4 + IL-13)] polarization (111). Inhibition of class I HDACs (e.g., HDAC3) lead to persistent activation of the NF-κB pathway *via* preventing deacetylation of RelA (p65) (112–114), which may induce the IKK-dependent expression of pro-inflammatory CXCL8 (IL-8) (115) or up-regulate CD47 to impair efferocytosis by M2-like macrophages (25, 116). Similarly, acetylation of STAT6 also suppresses M2 [M(IL-4)] polarization (117). Therefore, owe to such diversity of HDACs’ substrates, HDAC inhibition may have either anti- or pro-inflammatory activities, dependently upon which class or individual HDAC(s) are targeted more specifically as well as which substrate(s) are involved more preferentially (118).

## Conclusion and Perspectives

Although multiple clinical trials have recently shown promising benefits of anti-inflammatory agents or immunotherapies in AS-related cardiovascular diseases, several major challenges for targeting inflammation in AS remains to be addressed to achieve successful clinical translation of novel targets as well as their targeted agents or therapies (119). Of note, a paradigm shift of AS from an “inflammatory” disease to an “epigenetic” disorder is emerging (120). Fast-increasing evidence supports that the epigenetic machinery plays a central role in the regulation of inflammation. It has been widely accepted that high phenotypic plasticity and functional diversity of macrophages stem from their flexibility in reprogramming gene expression at the transcriptional level, a process primarily orchestrated by the epigenetic machinery (121). Among numerous DNA (as well as RNA) or histone epigenetic modifications and their epigenetic modifiers (including writer, eraser, and reader), an increasing number of them have been demonstrated to be associated with macrophage polarization and functions in inflammation or AS. Theoretically, they and many more candidates could be considered a potential therapeutic target for the treatment of AS. However, only a few therapeutic agents targeting these epigenetic modifications or their modifiers have been investigated thus far in the animal models of AS. Therefore, it remains to be defined whether this approach would be effective and safe in the treatment of patients with AS-related diseases. But, a caution should be taken in the development of their inhibitors as anti-inflammatory therapeutics, due to their diverse functions in macrophages as well as their “off-target” effects (e.g., those involving non-histone proteins). Moreover, there are extensive cross-talks between different epigenetic mechanisms (e.g., DNA methylation and histone PTMs), epigenetic regulation and TFs, or epigenetic reprogramming and metabolic rewiring in the regulation of macrophage polarization and functions (31, 122–125), which make targeting the epigenetic machinery even more challenging. To deal with this challenge, it is necessary to better understand the epigenetic mechanisms underlying macrophage-mediated non-resolving inflammation that drives AS (126). To this end, in addition to the traditional models for M1 or M2 polarization from M0 (resting) macrophages, which have been widely used to develop anti-inflammatory therapy, our observations from the GEP analysis raise a potential alternative model, in which M1 macrophages might be directly skewed to M2 phenotypes (repolarization) *via* epigenetic reprogramming of gene expression or silencing at the transcriptional level. Of note, this model has been supported by accumulating evidence from recent studies. For example, it has been found that histone lactylation might mediate the repolarization from M1 to M2 *via* a transition from glycolysis to oxidative phosphorylation (29, 30). Inhibition of NO production leads to repolarization of M1 to M2 *via* restoration of mitochondrial function that is impaired in M1, thus inhibiting AS (127). However, it is worth mentioning that M2 macrophages may have certain pro-AS properties. For example, CD163^+^ (M2-like) macrophages could promote intraplaque angiogenesis, vascular permeability, and leukocyte infiltration, leading to AS progression (128, 129). Nevertheless, the model proposed here could be useful to discover the epigenetic modifiers specific for governing the repolarization of macrophage from pro- (M1-like) to anti-inflammatory (M2-like) phenotype as novel therapeutic targets, which would hopefully change the game in the development of effective and safe anti-inflammatory therapy for IAADs like AS.

## Author Contributions

YD and FJ conceptualized, wrote, edited, and revised the manuscript. YD and HY gathered and analyzed the literature, and prepared the figures. YS and QL contributed to literature search and collection. All authors contributed to the article and approved the submitted version.

## Conflict of Interest

The authors declare that the research was conducted in the absence of any commercial or financial relationships that could be construed as a potential conflict of interest.

## Publisher’s Note

All claims expressed in this article are solely those of the authors and do not necessarily represent those of their affiliated organizations, or those of the publisher, the editors and the reviewers. Any product that may be evaluated in this article, or claim that may be made by its manufacturer, is not guaranteed or endorsed by the publisher.
